# AIDrugDesigner: a web server for drug-like molecules generation and optimization

**DOI:** 10.1093/bioinformatics/btag693

**Published:** 2026-09-21

**Authors:** Huimin Zhu, Renyi Zhou, Shiliang Zhang, Yifan Wu, Zhangli Lu, Dongsheng Cao, Min Li

**Affiliations:** Hunan Provincial Key Lab on Bioinformatics, School of Computer Science and Engineering, Central South University, Changsha, 410083, China; Hunan Provincial Key Lab on Bioinformatics, School of Computer Science and Engineering, Central South University, Changsha, 410083, China; Hunan Provincial Key Lab on Bioinformatics, School of Computer Science and Engineering, Central South University, Changsha, 410083, China; Hunan Provincial Key Lab on Bioinformatics, School of Computer Science and Engineering, Central South University, Changsha, 410083, China; Hunan Provincial Key Lab on Bioinformatics, School of Computer Science and Engineering, Central South University, Changsha, 410083, China; Xiangya School of Pharmacy, Central South University, Changsha, 410083, China; Hunan Provincial Key Lab on Bioinformatics, School of Computer Science and Engineering, Central South University, Changsha, 410083, China

## Abstract

**Motivation:**

Designing molecules with predefined properties remains a critical yet challenging task in drug discovery. Recent advances in deep learning have demonstrated strong potential in accelerating the identification of novel compounds with desired properties and activities. However, the implementation and running of these models often present difficulties due to varying environments and the requisite for additional coding competencies.

**Results:**

Here, we present AIDrugDesigner, an integrated web server for practical deep learning–driven drug design. The platform consists of two modules: generation and optimization. The generation module supports target-aware drug design, pharmacophore-guided, property-conditioned, and linker design from diverse inputs. The optimization module refines user-provided molecules with respect to drug-likeness, LogP, and synthetic accessibility. By integrating multiple generative strategies into a unified interface, AIDrugDesigner reduces the technical barrier for applying deep learning models in drug discovery. Systematic evaluations across multiple design tasks further demonstrate its versatility.

**Availability and implementation:**

The server is freely available at https://www.csuligroup.com/AIDrugDesigner.

## 1 Introduction

Hit identification and lead optimization are central stages of drug discovery. Multiple drug design strategies have been developed, each with distinct strengths and limitations. Structure-based drug design (SBDD) (Wang *et al.* 2018, Shanmuga Sundari *et al.* 2024) exploits the three-dimensional structure of target proteins to design molecules that bind specific pockets. This approach enables rational, target-directed molecular design. However, its effectiveness depends on the availability of high-resolution structural information. Proteins with dynamic or complex architectures complicate pocket identification, and SBDD is not applicable to targets lacking resolved structures. Ligand-based drug design (LBDD) (Kaushik *et al.* 2018) provides a complementary strategy when structural information is unavailable. It infers design principles from the structural similarity and activity profiles of known ligands, and is frequently applied when target structures are unresolved or during lead optimization. Unlike SBDD, LBDD does not require explicit structural knowledge of the protein. However, because it does not explicitly model target–ligand interactions, it may generate molecules with limited binding specificity or suboptimal target engagement. Fragment-based linker design (Bancet *et al.* 2020) represents another strategy that operates by connecting validated bioactive fragments into a unified scaffold. By restricting exploration to linker configurations between known functional fragments, this approach substantially reduces the search space relative to de novo molecular design. Importantly, fragment linking preserves key binding determinants, increasing the probability of maintaining biological activity. However, successful implementation often depends on accurate knowledge of fragment binding modes and spatial orientation. Inaccurate linker geometry can introduce conformational strain, reduce affinity, or compromise synthetic feasibility. Beyond target affinity, drug candidates must also satisfy additional physicochemical and pharmacokinetic properties, including stability, solubility, and bioavailability, to achieve adequate drug-likeness.

Recent advances in machine learning and molecular modelling have enabled the systematic computational implementation of these design strategies. These methods typically specialize in specific design paradigms and require substantial expertise in structural biology, medicinal chemistry, or computational modelling. The technical barriers associated with such tools limit their accessibility and may hinder broader adoption in drug discovery workflows. Accordingly, numerous computational platforms and biochemical knowledge-based tools have been developed. Structure-based platforms, such as OpenGrowth (Cheron *et al.* 2016), MolHyb (Wang *et al.* 2022a), MolAICal (Bai *et al.* 2021), and ReMODE (Wang *et al.* 2022b), require users to provide protein structural information and support structure-guided ligand design, often allowing fragment or pharmacophore constraints during optimization. Fragment-oriented tools, such as FragRep (Shan *et al.* 2020), integrate protein–ligand structural information to guide fragment replacement for ligand optimization. In parallel, molecular property optimization platforms, including ADMETopt (Yang *et al.* 2018), focus on refining predefined molecular scaffolds toward improved physicochemical and pharmacokinetic properties. Despite their utility, these tools generally operate within isolated design scenarios and lack seamless integration across multiple drug design strategies. In practice, rational drug discovery often integrates multiple design strategies. For example, molecules generated through structure-based design frequently require subsequent optimization of physicochemical properties to improve their drug-likeness. Integrated toolkits such as TorchDrug (Zhu *et al.* 2022) aggregate diverse deep learning modules for molecular representation, property prediction, de novo generation, and optimization. However, effective use of such frameworks requires substantial expertise in model configuration and access to significant computational resources. Collectively, a unified and user-accessible platform capable of supporting multiple drug design paradigms remains lacking.

To address the fragmentation of existing drug design tools, we developed AIDrugDesigner, a publicly accessible web platform for de novo molecular generation and optimization across multiple drug design scenarios. The server is organized into two functional modules. The Generation module supports structure-based, sequence-based, ligand-based, and constraint-guided molecular design using user-provided protein structures, molecular fragments, pharmacophore features, or predefined physicochemical properties. The Optimization module refines user-submitted molecules toward improved Quantitative Estimate of Drug-likeness (QED), lipophilicity (LogP), and Synthetic Accessibility (SA) within an integrated framework. Users can upload input structures or parameter specifications through a web interface and obtain generated or optimized molecular candidates with associated property predictions and visualizations. Systematic evaluations across multiple design tasks, as detailed in the Supplementary Information (SI), further demonstrate the versatility and robustness of the integrated framework and highlight its potential for practical drug discovery applications. By integrating multiple drug design approaches into an accessible online platform, AIDrugDesigner lowers the technical barriers associated with advanced deep learning models and facilitates practical applications in hit identification, scaffold hopping, and lead optimization.

### 2.1 Materials and methods

#### 2.1.1 Platform architecture and implementation

AIDrugDesigner was implemented in Python using the Django web framework. The server architecture consists of a front-end interface and a back-end computational engine. The front end was developed using HTML5, JavaScript and Bootstrap to enable interactive task submission, parameter configuration and results visualization. The back end integrates PyTorch for deep learning inference, RDKit (Landrum) and Open Babel (O’Boyle *et al.* 2011) for molecular structure handling and descriptor calculation, and scikit-learn for auxiliary property prediction. All generation and optimization models are deployed as independent inference modules and executed on GPU-enabled servers. Upon submission, each task is assigned a unique job identifier and processed in a queued execution system. After completion, results are stored temporarily on the server and can be downloaded in CSV format. Detailed usage instructions, parameter descriptions, and example input files are provided on the tutorial page and in Table S1; task management and data privacy policies are detailed in Supplementary Information Parts 2–3.

### 2.2 Molecular generation modules

AIDrugDesigner integrates multiple molecular generation strategies designed for distinct drug design scenarios (the overall workflow is shown in Fig. 1). All generated molecules are validated for chemical validity using RDKit prior to output. The output includes SMILES representations of candidate molecules, along with their physicochemical properties. The platform further provides interactive visualization of generated molecular structures together with associated properties. A complete list of calculated properties is provided in Table S2.

**Figure 1:**
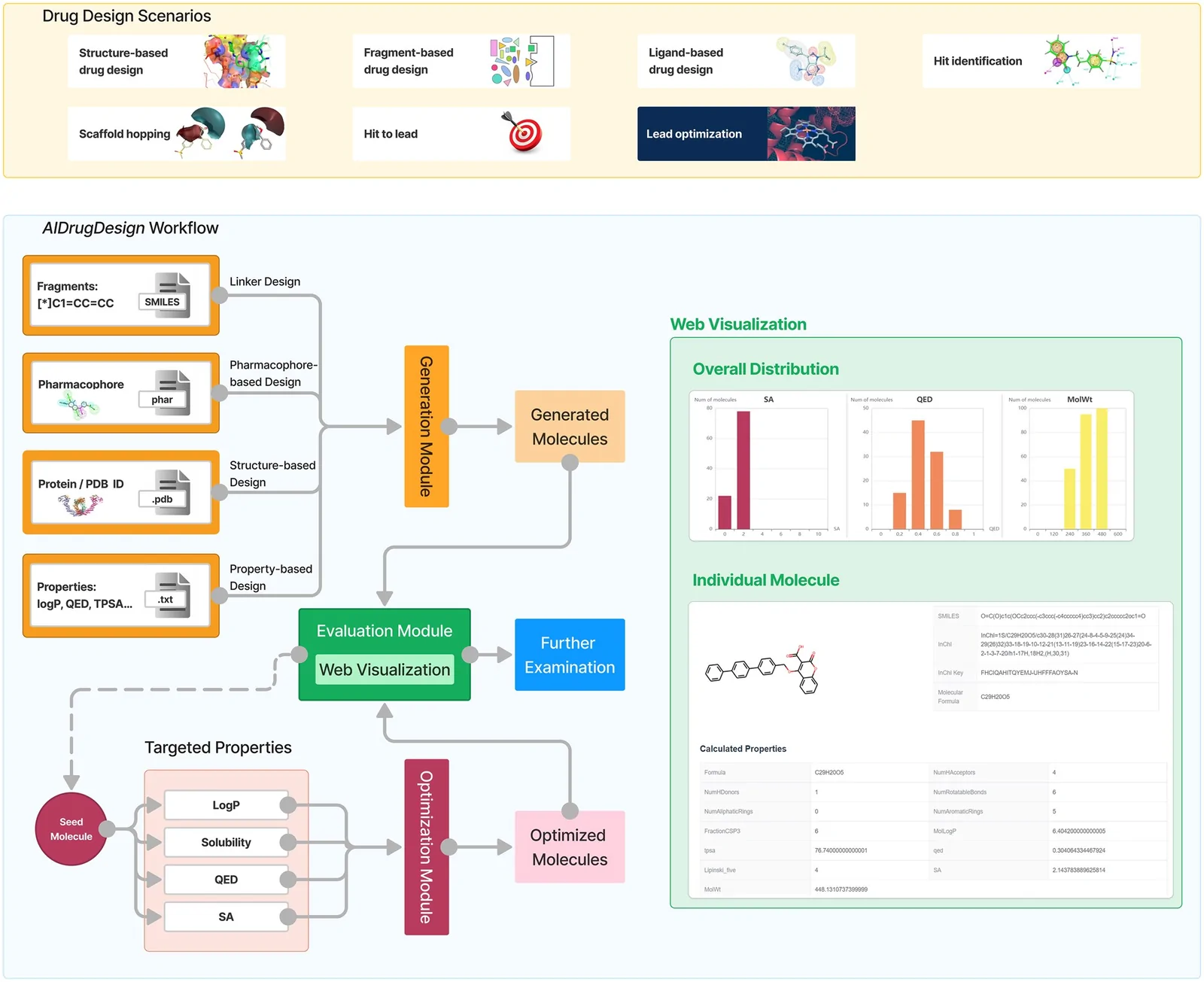
Workflow of AIDrugDesigner. Users can select either the generation or optimization module to design target molecules. The platform provides visualization of generated molecules and their property distributions.

#### 2.2.1 Structure-based generation

The SBDD-3D module provides an automated structure-based molecular generation pipeline (Li *et al.* 2021) that integrates pocket detection using Fpocket (Le Guilloux *et al.* 2009), enabling end-to-end target-guided design within a unified platform. Users can upload a PDB file or provide a PDB ID, and the server automatically identifies binding pockets and generates chemically valid molecules guided by the predicted pocket region.

#### 2.2.2 Sequence-based generation

The EncDecLM module generates target-specific molecules from protein sequence input. Users provide an amino acid sequence in FASTA format. The model follows a Transformer-based encoder–decoder architecture (Uludoğan *et al.* 2022). The model parameters were initialized using pretrained protein and chemical language models, respectively, and further trained on curated protein–ligand data.

#### 2.2.3 Pharmacophore-based generation

The Pharmacophore-based module generates molecules conditioned on predefined pharmacophore constraints (Zhu *et al.* 2023). Users upload a pharmacophore file containing spatial and physicochemical feature information. During generation, pharmacophore features are incorporated as structural constraints to guide molecular construction. The server returns chemically valid SMILES strings generated according to the specified pharmacophore constraints, together with associated molecular properties and structural visualizations.

#### 2.2.4 Property-based generation

The Property-based module implements a conditional recurrent neural network for physicochemical property-guided molecular generation (Kotsias *et al.* 2020). Users can specify desired value ranges for molecular properties, such as molecular weight, which are used as conditional inputs to guide molecular generation. The current implementation supports commonly used physicochemical properties, including MW, LogP, TPSA, HBA, HBD, and QED. The results include generated SMILES and their associated property values.

#### 2.2.5 Linker design

The Linker-design module is based on a graph-based generation framework that incorporates structural constraints during linker construction (Imrie *et al.* 2021). Users provide molecular fragments and specify desired linker properties, including linker length, hydrogen bond donor and acceptor counts. The model generates linkers that connect the input fragments while satisfying the specified constraints.

### 2.3 Molecular optimization modules

The optimization framework adopts a fragment-level modification strategy (Chen *et al.* 2021). Users submit a SMILES string of a molecule for optimization. The model identifies potential modification sites and performs single-fragment substitutions while preserving the core scaffold, enabling controlled structural refinement. The platform supports optimization of key molecular properties, including LogP, Solubility, QED, and Synthetic Accessibility (SA). For each task, candidates are retained only if the target property improves, non-target QED decreases by no more than 10%, non-target SA increases by no more than 10%, and the Morgan fingerprint-based Tanimoto similarity to the input molecule is at least 0.4.

### 2.4 Model evaluation

To evaluate the performance of the generation modules, we selected seven representative targets from the DOCKSTRING benchmark (García-Ortegón *et al.* 2022). The selected targets are described in Table S3. For Structure-based and Pharmacophore-based generation modules, molecular docking was performed to assess binding potential toward the corresponding targets. Docking grids were defined based on the binding pocket derived from the corresponding PDB structure. For comparison, experimentally reported active compounds retrieved from the ChEMBL database (Mendez *et al.* 2019) were also docked under the same settings. Detailed results are provided in Fig. S1. For Property-based and Linker-design modules, evaluation was conducted using conditional generative assessment metrics appropriate to each design scenario, with detailed results shown in Figs. S2 and S3. Specifically, physicochemical descriptors (including MW, LogP, TPSA, HBA, HBD, and QED) were calculated using RDKit, and compliance with user-specified constraints was assessed. For Linker design, structural integrity and constraint satisfaction were additionally verified, with detailed results provided in Fig. S3. For the Optimization modules, success rate and improvement were used as evaluation metrics. Success rate was defined as the proportion of generated molecules meeting the predefined optimization objective, while improvement quantified the magnitude of change relative to the input molecule. Detailed results are provided in Table S4. To provide a preliminary in silico assessment of the pharmacokinetic properties of molecules generated by the platform, ADMET profiles, including absorption, distribution, metabolism, excretion, and toxicity-related properties, were estimated using ADMETlab 3.0 (Fu *et al.* 2024). Detailed predicted results are provided in Fig. S4. The runtime performance of different molecular generation and optimization modules was systematically evaluated, with detailed statistics provided in Table S5.

## 3 Conclusion

We developed AIDrugDesigner, an integrated and publicly accessible web platform for the generation and optimization of drug-like molecules across diverse design scenarios. By consolidating multiple deep learning–based strategies within a unified interface, the platform reduces technical barriers and supports practical applications in de novo design, fragment-based design, ligand-based design, hit identification, scaffold hopping, and lead optimization. Interactive visualization and systematic evaluations further demonstrate the platform’s utility in facilitating streamlined and user-friendly molecular design workflows in drug discovery research.

## Supplementary Material

btag693_Supplementary_Data

## Code availability

The source code of AIDrugDesigner is available on GitHub at https://github.com/CSUBioGroup/AIDrugDesigner. The software version and associated files used in this study are archived on Figshare at https://doi.org/10.6084/m9.figshare.33475336. The AIDrugDesigner web platform is freely accessible at https://www.csuligroup.com/AIDrugDesigner.
