# Supplementary material for: AIDrugDesigner: a web server for drug-like molecules generation and optimization
Source: Bioinformatics. 2026 Sep 21;42(9):btag693. doi: 10.1093/bioinformatics/btag693 (PMC13623521; doi:10.1093/bioinformatics/btag693)
Supplement: Supplementary file 1 [file btag693_supplementary_data.zip › SI.docx]

# Supplementary Information

# AIDrugDesigner: a web server for drug-like molecules generation and optimization

Huimin Zhu^1^, Renyi Zhou^1^, Shiliang Zhang^1^, Yifan Wu^1^, Zhangli Lu^1^, Dongsheng Cao^2^, and Min Li^1^*

1 Hunan Provincial Key Lab on Bioinformatics, School of Computer Science and Engineering, Central South University, Changsha 410083, China

2 Xiangya School of Pharmacy, Central South University, Changsha 410083, China

* Corresponding author, limin@mail.csu.edu.cn

**Part 1 AIDrugDesigner Tutorial**

Tables S1 and S2 summarize the AIDrugDesigner workflow by listing the input parameters, scopes of applicability for each module, as well as the corresponding molecular descriptors generated. These tables provide users with practical guidance for selecting suitable design modes, preparing input data, and interpreting the generated results.

Table S1. Input Descriptions and Usage Instructions for AIDrugDesigner Modules.

|  | Methods | Description | Input Description and Applicable Scenarios | Web Page URL |
| --- | --- | --- | --- | --- |
| Generation | Structure-based | Generates molecules constrained to protein binding pockets, integrating pocket detection for user-friendly workflow. | PDB file or PDB ID of the target. Suitable for targets with reliable three-dimensional structures and well-defined binding pockets. | <https://www.csuligroup.com/AIDrugDesigner/#/generation/SBDD-3D> |
|  | Sequence-based | Integrates EncDecLM(Uludogan *et al.,* 2022) into AIDrugDesigner to generate target-specific molecules from protein sequences using pretrained RoBERTa(Filipavicius *et al.,* 2020) and ChemBERTa(Chithrananda *et al.,* 2020) models, enabling direct sequence-to-molecule design. | Protein sequence (FASTA). Suitable for target-specific molecule generation based on protein sequence information. | <https://www.csuligroup.com/AIDrugDesigner/#/generation/SequenceBased> |
|  | Phar-based | Generates molecules guided by user-defined pharmacophore features, ensuring compliance with spatial and chemical constraints. | A pharmacophore file is needed. Get it from the align-it tool or any other method. Suitable when key ligand–target interaction features or structure–activity relationships are available, such as in structure-based or ligand-based drug design. | <https://www.csuligroup.com/AIDrugDesigner/#/generation/phar-based> |
|  | Property-based | Conditional generative model based on recurrent neural networks, using the PhysChem Based (PCB)(Kotsias *et al.,* 2020) model to generate molecules conditioned on physicochemical descriptors such as logP, TPSA, MW, HBD, HBA, and QED. | Target ranges for MW, logP, TPSA, HBA, HBD, QED. Suitable for generating molecules with desired physicochemical or drug-like properties. | <https://www.csuligroup.com/AIDrugDesigner/#/generation/property-based> |
|  | Linker-based | Integrates GNN- and CNN-based linker design methods and explicitly incorporates desired properties—such as linker length, the number of hydrogen bond acceptors, and hydrogen bond donors—into the molecular generation process. | Molecular fragments and desired linker properties can be provided as input. Suitable when active fragments or binding moieties are known, such as in structure-based, ligand-based, or multi-target drug design. | <https://www.csuligroup.com/AIDrugDesigner/#/generation/linker-design> |
| Optimization | LogP | Optimizes molecular LogP using single-fragment modifications while retaining scaffold. | The SMILES of the molecule to be optimized. Suitable for active molecules requiring lipophilicity optimization while retaining the main scaffold. | <https://www.csuligroup.com/AIDrugDesigner/#/optimization/logP> |
|  | Solubility | Improves solubility by stepwise fragment-level modifications. Here, solubility optimization is operationally defined as reducing molecular lipophilicity. | The SMILES of the molecule to be optimized. Suitable for active molecules requiring reduced lipophilicity while retaining the main scaffold. | <https://www.csuligroup.com/AIDrugDesigner/#/optimization/hydrophilic> |
|  | QED | Optimize QED through single-site, fragment-level molecular modification while preserving the core scaffold. | The SMILES of the molecule to be optimized. Suitable for active molecules requiring improved drug-likeness through constrained molecular modification. | <https://www.csuligroup.com/AIDrugDesigner/#/optimization/QED> |
|  | synthetic accessibility (SA) | Optimize SA by modifying one fragment at a predicted disconnection site, preserving the main scaffold and ensuring controlled optimization with constrained molecular similarity. | The SMILES of the molecule to be optimized. Suitable for active molecules requiring improved synthetic accessibility while retaining molecular similarity. | <https://www.csuligroup.com/AIDrugDesigner/#/optimization/SA> |

Table S2. Output Information and Descriptions.

| Molecular descriptors | Description |
| --- | --- |
| SMILES | Text string encoding the molecular structure in a linear format. |
| InChI | Standardized textual identifier describing molecular structure. |
| InChIKey | Fixed-length hashed version of the InChI for database indexing. |
| Formula | Elemental composition of the molecule. |
| NumHAcceptors | Number of hydrogen bond acceptor atoms. |
| NumHDonors | Number of hydrogen bond donor atoms. |
| NumRotatableBonds | Count of rotatable single bonds reflecting molecular flexibility. |
| NumAliphaticRings | Number of non-aromatic ring systems. |
| FractionCSP3 | Proportion of sp³-hybridized carbons among total carbons. |
| MolLogP | Calculated lipophilicity (LogP). |
| TPSA | Topological polar surface area. |
| Lipinski_five | Indicator of compliance with Lipinski’s Rule of Five. |
| SA | Estimated synthetic accessibility score. |
| MolWt | Calculated molecular weight. |

**Part 2 Task Submission and Task Management**

To ensure stable access to the web server and efficient use of computational resources, AIDrugDesigner manages submitted jobs through a time-ordered queuing mechanism. Tasks are executed sequentially according to their submission time. After a task is submitted, users can track its status through the web interface. The task status is mainly divided into three stages: “Submitted successfully!”, indicating that the task has been successfully submitted; “Running”, indicating that the task is currently being processed and displayed when users access the task-specific result page; and the final result page, which is displayed once the task is completed. Users who provide an email address are notified when the results become available.

To balance computational cost, generation efficiency, and the need to provide a sufficient number of candidate molecules for downstream selection, each task supports the generation of up to 100 molecules, providing a sufficient set of candidate molecules for downstream screening and analysis.

**Part 3 Data retention and user privacy policy**

AIDrugDesigner accepts user-submitted input data, including molecular structures, protein sequences, pharmacophore files, and other task-related files, solely for the purpose of executing the computational task submitted by the user. Uploaded data and generated results are not used for redistribution, sharing, or any other secondary purpose.

To support result retrieval, task-related input files and generated results are temporarily stored on the server and automatically removed by a scheduled cleanup procedure after six months of retention. Each task is assigned a randomly generated, non-public task ID. Users access their results through the task-specific link provided after submission or through the same link delivered by email when an email address is supplied. Task-specific result pages are neither displayed nor indexed on public pages.

Communication between the computational backend and the web server is protected by key-based authentication, restricting the submission of task results to authorized backend services. Users are advised not to upload highly sensitive, confidential, or proprietary data unless they are authorized to do so and consider the stated temporary storage and scheduled deletion policy appropriate for their data.

**Part 4 Evaluation of the generation models**

To assess the performance of structure-based molecular design across diverse target classes, we selected one representative target from each of the seven functional classes defined in DOCKSTRING(García-Ortegón *et al.,* 2022) for testing. Detailed information was provided in Table S3.

Table S3 Targets and PDB Sets in the Validation of Structure-Based Molecular Design.

| Gene | PDB ID | Uniprot ID | Description |
| --- | --- | --- | --- |
| CDK2 | 4KD1 | P24941 | Cyclin-dependent kinase 2; aberrant activation is associated with cancer and certain neurodegenerative diseases. |
| PARP1 | 6I8M | P26446 | Poly (ADP-ribose) polymerase 1; an enzyme involved in DNA single-strand break repair and signal transduction, and a well-established oncology target. |
| PPARA | 1I7G | Q07869 | Peroxisome proliferator-activated receptor alpha; a nuclear receptor that regulates fatty acid oxidation and lipid metabolism. |
| BACE1 | 2IRZ | P56817 | Beta-secretase 1; responsible for the cleavage of amyloid precursor protein and implicated in Alzheimer’s disease. |
| DRD3 | 3PBL | P35462 | Dopamine receptor D3; involved in the regulation of cognition, emotion, and endocrine function. |
| CYP2C9 | 1OG5 | P11712 | Cytochrome P450 2C9; participates in the metabolism of various endogenous substrates, including fatty acids and steroids. |
| HSP90AA1 | 3HHU | P07900 | Heat shock protein 90 alpha family class A member 1; facilitates the maturation, structural stabilization, and functional regulation of specific client proteins. |

The structure-based SBDD-3D module was evaluated using the PDB(Burley *et al.,* 2017) complex structures of the seven representative targets listed in Table S3, with 10,000 independent sampling iterations performed for each target. The sequence-based EncDecLM module was assessed using the corresponding protein sequences of the same targets, generating 10,000 samples per sequence. For the pharmacophore-based (Phar-based) module, pharmacophore hypotheses corresponding to the targets listed in Table S3 were obtained from published studies and the ePharmaLib database(Moumbock *et al.,* 2021) for the targets in Table S3, followed by 10,000 independent sampling iterations per hypothesis. As SBDD-3D(Li *et al.,* 2021), EncDecLM, and the pharmacophore-based method are target-driven design strategies, molecular docking using AutoDock Vina(Trott and Olson 2010) was performed to evaluate the binding potential of the generated molecules toward their corresponding targets. For comparison, experimentally reported active compounds with activity values better than 100 μM were retrieved from the ChEMBL database(Bento *et al.,* 2014) and subjected to the same docking protocol.

**Figure S1** | Comparison of docking scores among molecules generated by the structure-based, sequence-based, and pharmacophore-based modules and active compounds retrieved from ChEMBL.

**Figure S2** | Comparison of physicochemical property distributions of property-conditioned generated molecules with the corresponding input conditions.

The property-based method was evaluated using activity data associated with the seven targets in Table S3. Active compounds with activity values better than 100 μM were retrieved from the ChEMBL database, and the mean values of key molecular descriptors—including LogP, TPSA, MW, HBD, HBA, and QED—were calculated for each target. These averaged descriptor values were used as conditional inputs for 10,000 independent sampling iterations per target. To assess conditional controllability, we compared the physicochemical properties of the generated molecules with their corresponding input conditions. As shown in Figure S2, the distributions of LogP, TPSA, MW, HBD, and HBA are centered around the specified target values, whereas QED exhibits a relatively broader distribution. Overall, most generated molecules satisfy the prescribed physicochemical constraints, demonstrating effective property control under the specified conditions.

The linker-based method was evaluated using the 321 fragment pairs from the PDBbind(Liu *et al.,* 2015) test set reported in the original DEVELOP(Imrie *et al.,* 2021) study. For our evaluation, 1,500 linkers were generated for each fragment pair, yielding 481,500 candidate molecules in total. To ensure a fair assessment, we compared the physicochemical property distributions of the generated molecules with those of molecules in the same PDBbind test set. Specifically, lipophilicity (LogP), hydrogen bond acceptors (HBA), hydrogen bond donors (HBD), and rotatable bonds were analyzed using empirical cumulative distribution functions (ECDFs). As shown in Figure S3, the ECDF curves of the generated molecules closely align with those of the PDBbind test set, indicating comparable overall physicochemical profiles. In addition, we performed a further analysis of the linker moieties extracted from the generated molecules and compared them with linkers from the PDBbind test set using the same ECDF-based evaluation. The resulting distributions show substantial overlap, suggesting that the proposed method effectively captures the structural and physicochemical characteristics of linkers observed in real datasets.

**Figure S3** | ECDF comparison of physicochemical property distributions between generated molecules and the PDBbind test set, as well as between generated linkers and PDBbind linkers.

## Part 5 Evaluation of the Optimization Module

To evaluate the molecular optimization module of the AIDrugDesigner platform, we utilized 800 publicly available test molecules from Modof(Chen *et al.,* 2021). Optimization success rate and the extent of property improvement were used as the primary evaluation metrics. For each test molecule, 10 independent sampling runs were performed to generate optimized candidates. “Success rate (%)” denotes the proportion of generated candidates whose target property was improved in the desired optimization direction relative to the corresponding original molecule. “Improvement” represents the mean change in the target property relative to the original molecule, with the direction defined according to the corresponding optimization objective. “std” represents the standard deviation of these property changes. “Similarity” denotes the structural similarity between the original molecules and their optimized counterparts.

As shown in Table S4, substantial improvements were achieved for most optimized properties, including LogP, solubility, and SA, under a single-fragment optimization setting in which only one molecular fragment was modified at a time. This design strategy was adopted to better mimic the stepwise optimization process commonly used in medicinal chemistry, thereby enhancing the interpretability and synthetic feasibility of the optimized molecules. In contrast, optimization of QED showed relatively limited improvement, likely due to the high baseline QED value (mean = 0.75) of the test set, which constrained further enhancement.

Table S4 Overall Performance of the Molecule Optimization Module.

| Optimizing property | Improvement ± std | Success rate | Similarity ± std |
| --- | --- | --- | --- |
| Optimizing LogP | 3.406±1.76 | 99.9% | 0.528±0.115 |
| Optimizing Solubility | 1.93±1.44 | 96.0% | 0.529±0.115 |
| Optimizing QED | 0.078±0.052 | 87.5% | 0.529±0.115 |
| Optimizing SA | 0.474±0.455 | 85.4% | 0.491±0.137 |

**Part 6 In Silico ADMET Evaluation**

To assess the pharmacokinetic (absorption, distribution, metabolism, and excretion; ADME) and toxicity profiles of molecules generated by the AIDrugDesigner platform, 1,000 molecules were randomly sampled for each module. ADMET properties were predicted using ADMETlab 3.0(Fu *et al.,* 2024), and the proportion of molecules satisfying each criterion was determined based on the tool’s recommended threshold values. As shown in Figure S4, a high proportion of generated molecules met the predefined pharmacokinetic and toxicity criteria, indicating that the platform is capable of producing molecules with favourable drug-like properties.

**Figure S4** | Distribution of predicted ADMET properties for AIDrugDesigner-generated molecules. Dashed lines indicate the recommended thresholds for each property; upward and downward arrows denote properties for which higher or lower values are preferred, respectively. Evaluated properties include physicochemical and synthetic parameters (TPSA, MW, nHD, nHA, SA), permeability-related metrics (PAMPA, BBB, F(20%)), metabolic and toxicity predictors (CYP2C9, hERG, ROA), and plasma clearance (CL-plasma). All ADMET profiles are based on in silico predictions from ADMETlab 3.0.

**Part 7 Platform Runtime Analysis**

Table S5 Runtime Statistics of Different Molecular Generation and Optimization Modules.

| Methods | Runtime for tasks targeting 30 valid molecules(s) | Runtime for tasks targeting 50 valid molecules(s) | Runtime for tasks targeting 100 valid molecules(s) |
| --- | --- | --- | --- |
| Structure-based | 779.61 ± 276.78 | 905.35 ± 350.83 | 1076.90 ± 426.12 |
| Sequence-based | 21.86 ± 2.04 | 25.57 ± 4.65 | 26.29 ± 2.56 |
| Phar-based | 674.92 ± 1485.56 | 998.90 ± 2201.71 | 2034.19 ± 4487.89 |
| Property-based | 106.56 ± 31.87 | 174.12 ± 56.85 | 340.87 ± 106.28 |
| Linker-based | 35.35 ± 8.75 | 56.61 ± 13.22 | 110.90 ± 27.79 |
| LogP | 209.90 ± 362.15 | 517.17 ± 792.06 | 565.73 ± 597.05 |
| Solubility | 674.36 ± 680.14 | 1173.35 ± 873.03 | 1876.60 ± 785.11 |
| QED | 379.93 ± 453.73 | 818.64 ± 897.51 | 992.83 ± 833.99 |
| SA | 379.86 ± 526.83 | 697.08 ± 887.78 | 1016.54 ± 814.99 |

Table S5 summarizes the wall-clock runtime, expressed as the mean ± standard deviation in seconds, for molecular generation and optimization tasks configured with target output sizes of 30, 50, and 100 valid molecules. All runtime benchmarks were conducted independently using a single NVIDIA GeForce RTX 2080 Ti GPU with 11 GB of GPU memory. The server ran NVIDIA driver version 440.33.01 and CUDA 10.2. This analysis was performed to characterize the practical runtime requirements of the unified molecular generation platform under representative task settings.

The structure-based and sequence-based modules were evaluated using the seven protein targets listed in Table S3. The pharmacophore-based module was assessed using pharmacophore constraints containing three to eight features. The property-based, linker-based, and molecular optimization modules, including LogP, Solubility, QED, and SA optimization, were evaluated using 100 molecules randomly sampled from the PDBbind dataset. For property-based generation, the target property values were calculated from the sampled molecules and used as generation conditions. For linker-based generation, fragment pairs were derived from the same molecular set. For each molecular optimization task, one of the 100 sampled molecules was used as the starting structure, resulting in 100 independent optimization tasks for each optimization module.

Runtime varied across modules because of differences in model architecture, input characteristics, constraint complexity, sampling efficiency, and the number of invalid molecules produced before termination. For the molecular optimization modules, differences in the physicochemical properties of the starting molecules resulted in substantial runtime variation among individual tasks. The pharmacophore-based module was particularly sensitive to constraint complexity. For example, tasks containing eight pharmacophore features required an average of 3702.03 s to generate 30 molecules, compared with 101.37 s for tasks containing three to seven features. This difference explains the relatively large standard deviations observed for this module.

Each task was terminated when either the target number of valid molecules was obtained or the predefined maximum sampling limit was reached. Therefore, in a small number of highly constrained cases, the requested number of valid molecules was not fully obtained before termination. The reported values consequently represent the runtime of tasks configured with target output sizes of 30, 50, and 100 valid molecules, rather than guaranteed completion times in every individual case.

**References**

Bento AP, Gaulton A, Hersey A *et al.* The chembl bioactivity database: An update. *Nucleic acids research* 2014;**42**:D1083-D90.

Burley SK, Berman HM, Kleywegt GJ *et al.* Protein data bank (pdb): The single global macromolecular structure archive. *Protein crystallography: methods and protocols* 2017;**1607**:627-41.

Chen Z, Min MR, Parthasarathy S *et al.* A deep generative model for molecule optimization via one fragment modification. *Nature Machine Intelligence* 2021;**3**:1040-9.

Chithrananda S, Grand G, Ramsundar B. Chemberta: Large-scale self-supervised pretraining for molecular property prediction. *arXiv preprint arXiv:2010.09885* 2020.

Filipavicius M, Manica M, Cadow J *et al.* Pre-training protein language models with label-agnostic binding pairs enhances performance in downstream tasks. *arXiv preprint arXiv:2012.03084* 2020.

Fu L, Shi S, Yi J *et al.* Admetlab 3.0: An updated comprehensive online admet prediction platform enhanced with broader coverage, improved performance, api functionality and decision support. *Nucleic acids research* 2024;**52**:W422-W31.

García-Ortegón M, Simm GN, Tripp AJ *et al.* Dockstring: Easy molecular docking yields better benchmarks for ligand design. *Journal of chemical information and modeling* 2022;**62**:3486-502.

Imrie F, Hadfield TE, Bradley AR *et al.* Deep generative design with 3d pharmacophoric constraints. *Chemical science* 2021;**12**:14577-89.

Kotsias P-C, Arus-Pous J, Chen H *et al.* Direct steering of de novo molecular generation with descriptor conditional recurrent neural networks. *Nature Machine Intelligence* 2020;**2**:254-65.

Li Y, Pei J, Lai L. Structure-based de novo drug design using 3d deep generative models. *Chemical science* 2021;**12**:13664-75.

Liu Z, Li Y, Han L *et al.* Pdb-wide collection of binding data: Current status of the pdbbind database. *Bioinformatics* 2015;**31**:405-12.

Moumbock AF, Li J, Tran HT *et al.* Epharmalib: A versatile library of e-pharmacophores to address small-molecule (poly-) pharmacology. *Journal of Chemical Information and Modeling* 2021;**61**:3659-66.

Trott O, Olson AJ. Autodock vina: Improving the speed and accuracy of docking with a new scoring function, efficient optimization, and multithreading. *Journal of computational chemistry* 2010;**31**:455-61.

Uludogan G, Ozkirimli E, Ulgen KO *et al.* Exploiting pretrained biochemical language models for targeted drug design. *Bioinformatics* 2022;**38**:ii155-ii61.
